# PDPN+ cancer‐associated fibroblasts enhance gastric cancer angiogenesis via AKT/NF‐κB activation and the CCL2‐ACKR1 axis

**DOI:** 10.1002/mco2.70037

**Published:** 2025-01-06

**Authors:** Zhenxiong Zhao, Hui Sun, Yingxue Liu, Yanqiu Zhang, Xin Wang, Xu Wang, Cong Tan, Shujuan Ni, Weiwei Weng, Meng Zhang, Lei Wang, Dan Huang, Wenchao Gu, Jinjia Chang, Weiqi Sheng, Mi‐die Xu

**Affiliations:** ^1^ Department of Gastric Surgery Fudan University Shanghai Cancer Center Shanghai China; ^2^ Department of Oncology Shanghai Medical College, Fudan University Shanghai China; ^3^ Department of Pathology Fudan University Shanghai Cancer Center Shanghai China; ^4^ Institute of Pathology Fudan University Shanghai China; ^5^ Department of Endoscopy Fudan University Shanghai Cancer Center Shanghai China; ^6^ Department of Artificial Intelligence Medicine, Graduate School of Medicine Chiba University Chiba Japan; ^7^ Department of Medical Oncology Fudan University Shanghai Cancer Center Shanghai China

**Keywords:** angiogenesis, cancer‐associated fibroblasts, CCL2, gastric cancer, PDPN

## Abstract

Cancer‐associated fibroblasts (CAFs) are intrinsic components of the tumor microenvironment that promote cancer progression and metastasis. Through an unbiased integrated analysis of gastric tumor grade and stage, we identified a subset of proangiogenic CAFs characterized by high podoplanin (PDPN) expression, which are significantly enriched in metastatic lesions and secrete chemokine (CC‐motif) ligand 2 (CCL2). Mechanistically, PDPN(+) CAFs enhance angiogenesis by activating the AKT/NF‐κB signaling pathway. The canonical NF‐κB signaling protein P65 binds to the promoter region of CCL2, inducing its expression. Additionally, we found that CCL2 interacts with its nonclassical receptor ACKR1 (expressed on endothelial cells) to exert its proangiogenic effects. Furthermore, the disruption of CCL2‐ACKR1 communication via a CCL2 neutralizing antibody or the inhibition of AKT signaling transduction using AKT inhibitors effectively suppressed tumor growth. Together, this study elucidates the mechanism by which PDPN(+) CAFs promote angiogenesis, providing a deeper understanding of the molecular processes underlying CAF‐mediated angiogenesis and suggesting potential therapeutic targets for gastric cancer treatment.

## Graphical abstract

The Akt/NF‐κB pathway was significantly activated in podoplanin (PDPN)(+) cancer‐associated fibroblasts (CAFs). P65 directly bind to the chemokine (CC‐motif) ligand 2 (CCL2) promoter, thereby increasing the CCL2 transcription and secretion in CAFs; CCL2, which was transcriptional activated by P65 in PDPN(+) CAFs, sustains tumor angiogenesis by interacting with ACKR1 and activating PI3K/AKT signaling in endothelial cells.

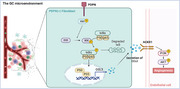

## 1 INTRODUCTION

Gastric cancer (GC) is among the most common malignancies in China, accounting for nearly 50% of the world's new cases and deaths associated with this disease.^1^ This high incidence presents a significant challenge to disease control efforts, making it a critical focus for cancer prevention and treatment.^2^ Angiogenesis plays a vital role in tumor growth and progression.^3^ However, clinical outcomes with current vascular endothelial growth factor receptor (VEGFR) inhibitors do not always yield favorable results. Although the anti‐VEGFR‐2 antibody Ramucirumab and the selective VEGFR‐2 tyrosine kinase inhibitor Apatinib have shown significant survival benefits in later lines of treatment, Ramucirumab did not prolong survival in randomized controlled phase III trials for both resectable and advanced disease.^4^ Similarly, a phase III randomized trial failed to confirm the efficacy of Apatinib in a global population.^5^ Given these challenges, there is an urgent need to identify new VEGF‐independent molecules and develop more effective antiangiogenesis therapies. Such therapies could potentially be combined with anti‐VEGF treatments to enhance patient outcomes. In diseases associated with abnormal angiogenesis, such as cancer, the activation of the PI3K/Akt signaling pathway promotes angiogenesis through various mechanisms, including the regulation of survival, proliferation, and migration of endothelial cells (ECs) to facilitate the formation of new blood vessels.^6^, ^7^ This makes the PI3K/Akt pathway a potential therapeutic target.

The tumor microenvironment (TME) is a complex ecosystem consisting of tumor cells, the surrounding extracellular matrix, blood vessels, and immune cells. Cancer‐associated fibroblasts (CAFs) play a crucial role in maintaining TME stability and promoting tumor development.^8^ CAFs acidify the cytoplasmic environment by altering intracellular signaling pathways, which influences the proliferation, invasion, metastasis and chemotherapeutic resistance of GC cells.^9^ Additionally, CAFs enhance the malignant behaviors of tumor cells by facilitating angiogenesis and increasing vascular permeability within the TME.^10^ For instance, CAFs secrete various cytokines and growth factors, including vascular endothelial growth factor A (VEGFA), platelet‐derived growth factor C (PDGFC), and fibroblast growth factor 2 (FGF2), which stimulate neovascularization.^11^, ^12^, ^13^, ^14^ Furthermore, the CAFs‐derived regulatory molecular lncSNHG5 promotes angiogenesis and vascular permeability by enhancing P38 MAPK signaling in ECs.^15^ Therefore, understanding the functional mechanism of CAFs in the TME could provide valuable insights for designing targeted therapies against GC.

The heterogeneity of CAFs is a critical factor in the development of targeted therapies.^16^ Due to the complexity and diversity of the TME, the expression and function of CAFs can vary significantly across different tumor types.^17^ This variability can render therapies targeting a single CAF subset potentially ineffective. Therefore, exploring the heterogeneous characteristics of CAFs in specific tumor types, identifying subsets associated with malignant phenotypes, analyzing the procancer effects within the TME, and pinpointing downstream molecular targets are essential for advancing therapeutic strategies. A deeper understanding CAFs heterogeneity will enable the development of more personalized and precise treatments, improving efficacy while minimizing unnecessary side effects. The membrane protein podoplanin (PDPN) is a well‐known cancer‐promoting molecule. Previously, we reported that PDPN(+) CAFs can enhance the metastasis of GC cells by secreting Periostin.^18^ By mediating the activation of ezrin, PDPN can regulate the viability, migration, and invasion of GC cells, but inhibited their apoptosis.^19^ Recently, it has been reported that PDPN(+) CAFs promote angiogenesis in colorectal cancer by activating signal transducer and activator of transcription 3 (STAT3) signaling pathway in ECs.^20^ However, the relationship between PDPN(+) CAFs and angiogenesis in GC remains unknown.

In this study, we specifically demonstrate the overexpression of PDPN in proangiogenic CAFs (anCAFs) within GC. We investigate the underlying mechanism, revealing that PDPN plays a critical role in angiogenesis by promoting the secretion of chemokine (CC‐motif) ligand 2 (CCL2) from CAFs. This secretion is recognized by the surface receptor ACKR1 on ECs, leading to the activation of the PI3K/AKT signaling pathway in these cells. Additionally, we found that the Akt/NF‐κB pathway was significantly upregulated in PDPN(+) CAFs compared to PDPN(−) CAFs. P65 directly binds to the CCL2 promoter, thereby increasing CCL2 transcription and secretion in CAFs. Furthermore, we unveil the clinical significance of the PDPN/CCL2 axis, providing insights into its proangiogenic role in the progression of GC and suggesting potential novel strategies for antiangiogenic therapy.

## 2 RESULTS

### 2.1 Elevated PDPN levels in patient‐derived CAFs correlate with tumor angiogenesis in GC

**FIGURE 1:**
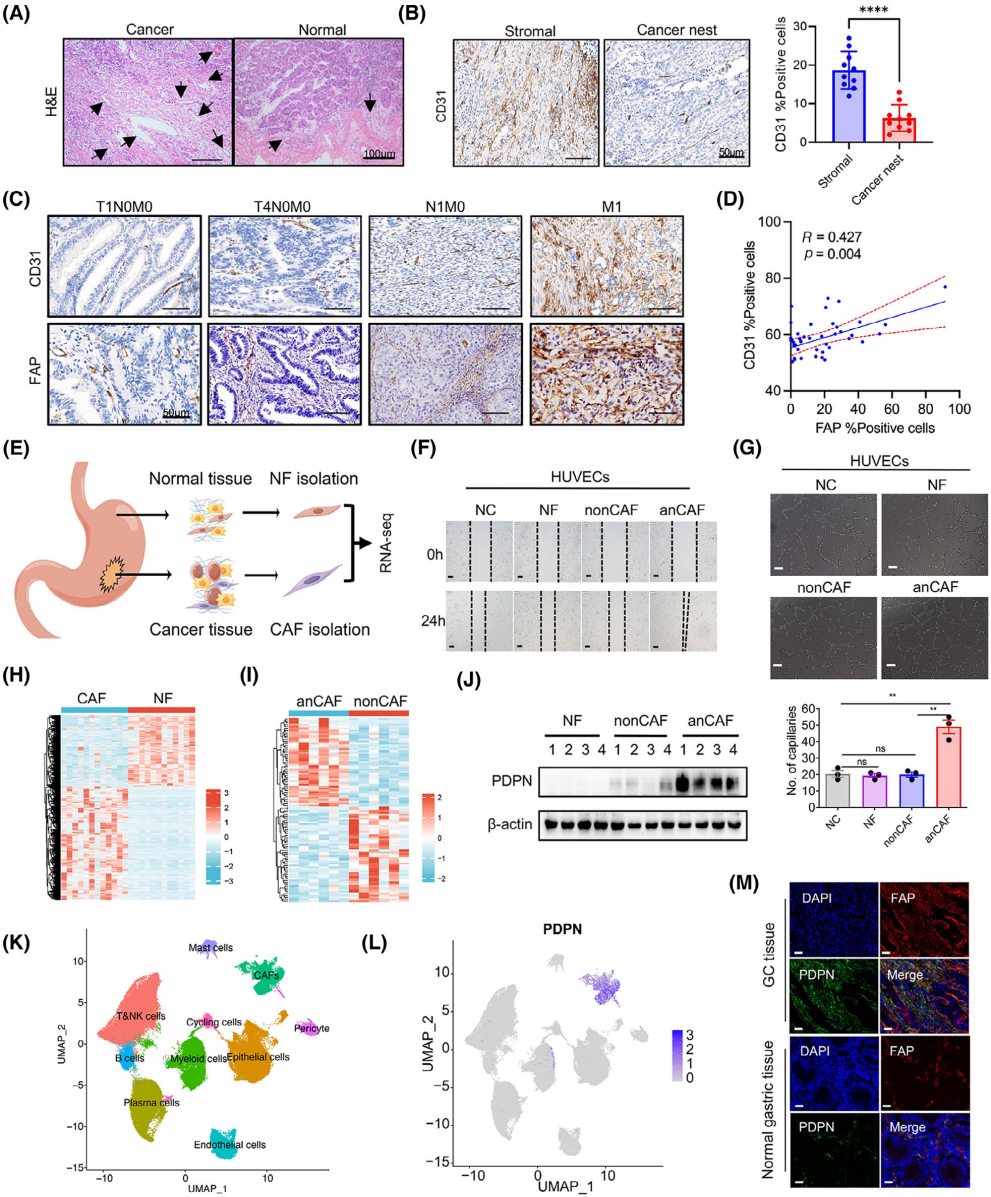
Elevated podoplanin (PDPN) levels in patient‐derived cancer‐associated fibroblasts (CAFs) correlate with tumor angiogenesis in gastric cancer (GC). (A) Hematoxylin and eosin (H&E) staining to show vascular structures within the stroma of both cancer and adjacent normal tissues. Scale bar, 100 µm. (B) Left panel: Immunohistochemical analysis of CD31 illustrating the distribution of vascular structures within the cancer nest and stroma regions. Right panel: Quantitative analysis of the percent of CD31‐positive cells, statistically evaluating the vascular density across the examined regions. Scale bar, 50 µm. (C) Immunohistochemical analysis of CD31 and fibroblast activation protein (FAP; a CAF marker) expressions in GC tissue samples with different infiltration depths, lymph node metastasis status, and distant metastasis status. Scale bar, 50 µm. (D) Positive correlation between the percent of FAP‐ and CD31‐positive cells in GC tissue samples, as determined by Pearson's correlation test. (E) Schematic representation of the workflow for sample preparation from GC patients for fibroblasts isolation and RNA‐sequencing. The scheme was drawn by Figdraw (www.figdraw.com). (F, G) Wound healing assay (F) and tube formation assay (G) performed on human umbilical vein endothelial cells (HUVECs) cultured in conditioned medium (CM) derived from normal fibroblast (NF), nonCAFs, and anCAFs (scale bar = 100 µm; **p < 0.01). (H, I) Heatmap showing significantly dysregulated genes in CAFs versus NF (H; *n* = 12) and in anCAFs versus nonCAFs (I; *n* = 6). (J) Western blots results showed the PDPN expression levels in NF, nonCAFs and anCAFs (*n* = 4). (K) Uniform manifold approximation and projection (UMAP) of cells representing 10 unique cell states color‐coded by their corresponding cell lineage or subtype. Each dot in the UMAP represents a single cell. (L) UMAP plot displaying PDPN expression across the total cell population. (M) Representative immunofluorescent staining for 4',6‐diamidino‐2‐phenylindole (DAPI), FAP, and PDPN in clinical GC and normal gastric tissues (scale bar = 40 µm).

Increasing evidence indicates that CAFs play a vital role in promoting angiogenesis across various cancers. To investigate whether CAFs contribute to angiogenesis in GC, we analyzed clinical samples. Hematoxylin and eosin (H&E) staining revealed numerous vessel‐like structures in the gastric tumor stroma compared to the nontumor stromal area (Figure 1A). This finding was further validated by CD31 staining, a specific biomarker for vascular ECs (Figure 1B). Consistent with our previous findings, the content of CAFs, as indicated by the immunostaining of fibroblast activation protein (FAP), was significantly higher in GC samples than in intraepithelial neoplasia samples and normal gastric samples.^21^ This suggests a potential role of CAFs in GC angiogenesis.

We further evaluated CD31 immunohistochemical staining using the same series of tissue microarray (TMA) slides previously employed for FAP expression detection. The representative figure depicts the expression of CD31 and FAP in GC tissue samples with varying infiltration depths, lymph node metastasis, and distant metastasis status. It appears that the expression of both CD31 and FAP increased with the progression and metastasis of GC (Figure 1C). In cancer samples, a positive correlation was noted between the percentage of FAP‐positive cells and the percentage of CD31‐positive cells (Figure 1D), suggesting that CAFs likely promote gastric tumor angiogenesis.

To investigate whether CAFs influence the angiogenic behavior of vascular ECs, we isolated CAFs and normal fibroblasts (NFs) from tumor and normal gastric tissues, respectively (Figure 1E). We then cocultured patient‐derived CAFs with human umbilical vein endothelial cells (HUVECs). As illustrated in Figure 1F,G, CAFs from certain GC patients significantly enhanced the migration potential and tube formation of HUVECs, while other CAFs and NFs did not demonstrate this capability. These findings suggest that CAFs are heterogeneous and functionally diverse in tumor progression, with a subset of proangiogenic CAFs (anCAFs) present in the GC stroma.

To identify the critical genes in anCAFs, we performed bulk RNA sequencing on anCAFs, nonproangiogenic CAFs (nonCAFs), and their paired normal gastric fibroblasts (NFs). In the comparison between CAFs and NFs groups (*n* = 12), we detected 1198 genes that were upregulated and 758 that were downregulated (Figure 1H). In the analysis of anCAFs versus nonCAFs groups (*n* = 6), there were 47 upregulated genes and 51 downregulated genes identified (False Discovery Rate < 0.05, |log2 fold change (FC)| > 1; Figure 1I). Moreover, gene ontology analysis results revealed that angiogenesis‐associated genes were significantly upregulated in primary CAFs (Figure S1A). We further selected 23 genes that were significantly elevated in anCAFs compared to nonCAFs and NFs (Figure S1B). Among them, four membrane protein‐encoding genes—DPCR1, PDPN, ITGA11, and PRTG—were identified as potential cell‐surface markers for defining the anCAFs subset. Notably, PDPN was the only gene with both mRNA (Figure S1C) and protein (Figure 1J) upregulated in anCAFs compared to nonCAFs and/or NFs.

Additionally, the analysis of published single‐cell RNA sequencing (scRNA‐seq) data from GC samples^22^ allowed us to annotate various cell types, including 24,854 epithelial cells, 51,410 T&NK cells, 5544 B cells, 19,045 plasma cells, 14,198 myeloid cells, 8276 fibroblast cells, 6992 ECs, 2694 pericytes, and 1528 cycling cells from GC, encompassing different clinical stages and peritoneal metastatic lesions (Figure 1K). PDPN expression was predominantly observed in CAFs rather than in other cell types within the TME (Figure 1L). Coimmunofluorescence staining for PDPN and FAP in GC tissues and normal gastric tissues further demonstrated the abundant PDPN protein expression in stromal CAFs (Figure 1M). These findings suggest that stromal fibroblasts may play a role in angiogenesis in GC, and the expression and function of PDPN in stromal fibroblasts warrant further investigation.

### 2.2 Pseuodotime analysis indicates the potential origin of PDPN(+) CAFs subpopulations in GC

**FIGURE 2:**
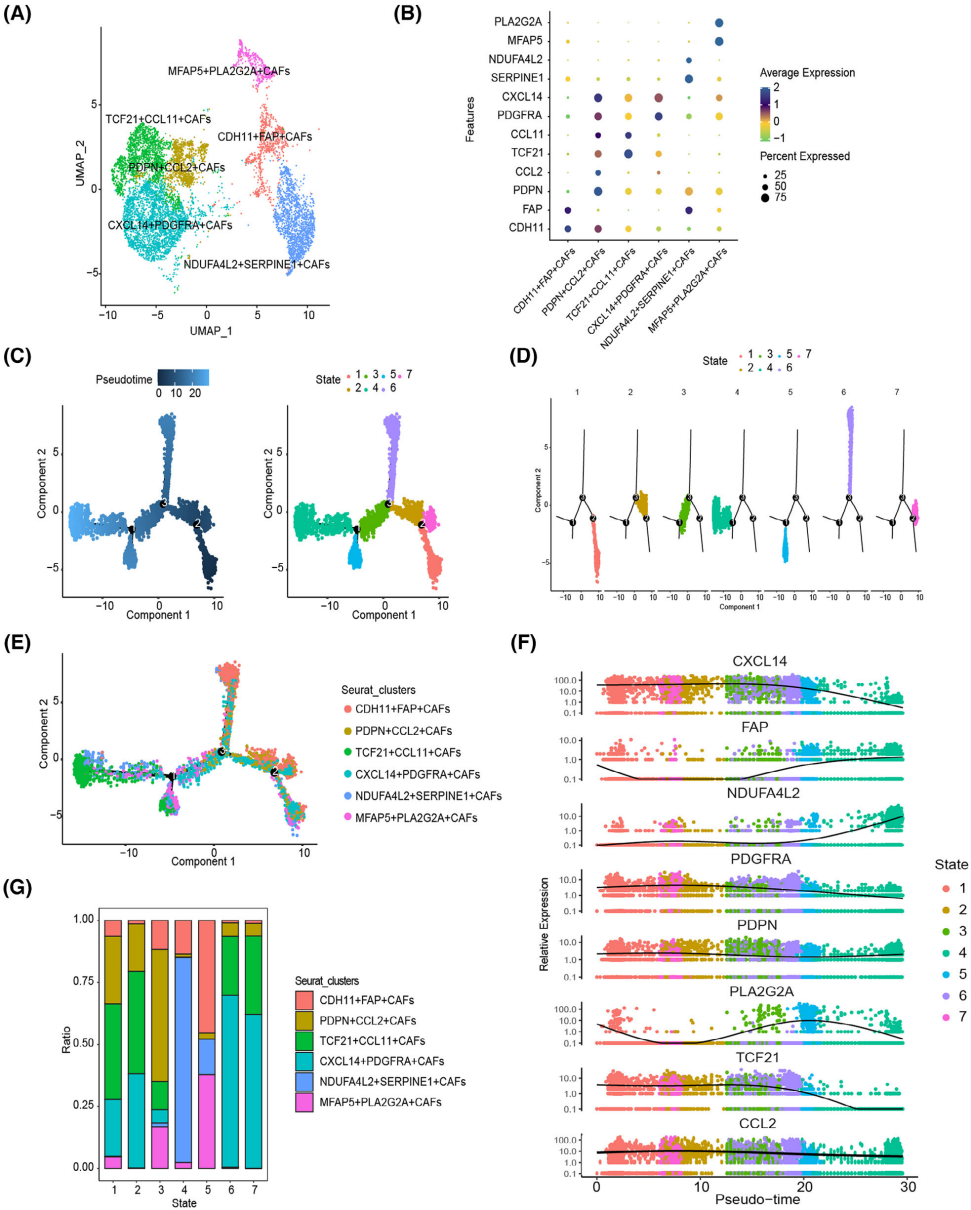
The heterogeneity within fibroblast subpopulations. (A) Uniform manifold approximation and projection (UMAP) visualization of fibroblast subclusters. Each dot represents a single cell. (B) Marker genes from each fibroblast subclusters are illustrated using a dot plot. (C–F) Pseudotime cell trajectories analysis using Monocle 2 to investigate the dynamic changes in fibroblast cells. Visualization in pseudotime (C), state (D), facet wrap by state (E), and Seurat clusters (F). Pseudotime trajectory analysis results indicated activation of fibroblast activation protein (FAP) and NDUFA4L2 over the pseudotime, while the CXCL14, TCF21, and WNT5A lost their expression during the process. (G) Stack graph showed the ratio of fibroblast subsets in each state.

To elucidate the origins of PDPN(+) CAFs in GC, we conducted pseudotime analysis on 8276 fibroblast cells, identifying six distinct subclusters based on unique gene expression profiles (Figure 2A,B). Notably, *FAP‐α*, a well‐established marker for activated fibroblasts, was highly expressed in two subclusters: CDH11^+^FAP^+^CAFs and NDUFA4L2^+^SEPPINE1^+^CAFs (Figure 2B). Additionally, we identified another subcluster characterized by the expression of PDPN and CCL2, a previously reported mediator of angiogenesis.^23^ We termed this group PDPN^+^CCL2^+^CAFs. The remaining three subclusters exhibited distinct gene expression profiles, characterized by *TCF21*, *CCL11*, *PDGFRA*, *CXCL14*, *MFAP5*, and *PLA2G2A*, leading us to designate them as TCF21^+^CCL11^+^CAFs, CXCL14^+^PDGFRA^+^CAFs, and MFAP5^+^PLA2G2A^+^CAFs, respectively (Figure 2B).

To explore the developmental trajectories of these six subpopulations, we employed the Monocle 2 algorithm, leveraging their transcriptional similarities.^24^ The results indicated that the initial state (states 1 and 2) was predominantly composed of TCF21^+^CCL11^+^CAFs and CXCL14^+^PDGFRA^+^CAFs, characterized by signature genes such as CXCL14 and TCF21. In contrast, the end state (state 4) was dominated by NDUFA4L2^+^SEPPINE1^+^CAFs, which exhibited signature genes of FAP and NDUFA4L2, suggesting a potential progression from an inactive to an activated state (Figure 2C–F). Increased CCL2 expression, recognized as a marker of activated fibroblasts,^25^ was notably enriched in state 3, the transition state, where we observed a significant presence of PDPN^+^CCL2^+^CAFs (Figure 2G). Enrichment analysis revealed that highly expressed genes in PDPN^+^CCL2^+^CAFs are involved in biological processes such as extracellular matrix organization, focal adhesion, and vasculature development (Figure S1D). This finding further underscores the robustness of our trajectory analysis. Collectively, these results suggest that PDPN(+)CAFs may originate from TCF21^+^CCL11^+^CAFs and CXCL14^+^PDGFRA^+^CAFs subpopulations, undergoing a progressive activation process.

### 2.3 PDPN(+)CAFs‐induced angiogenesis in vitro and in vivo

**FIGURE 3:**
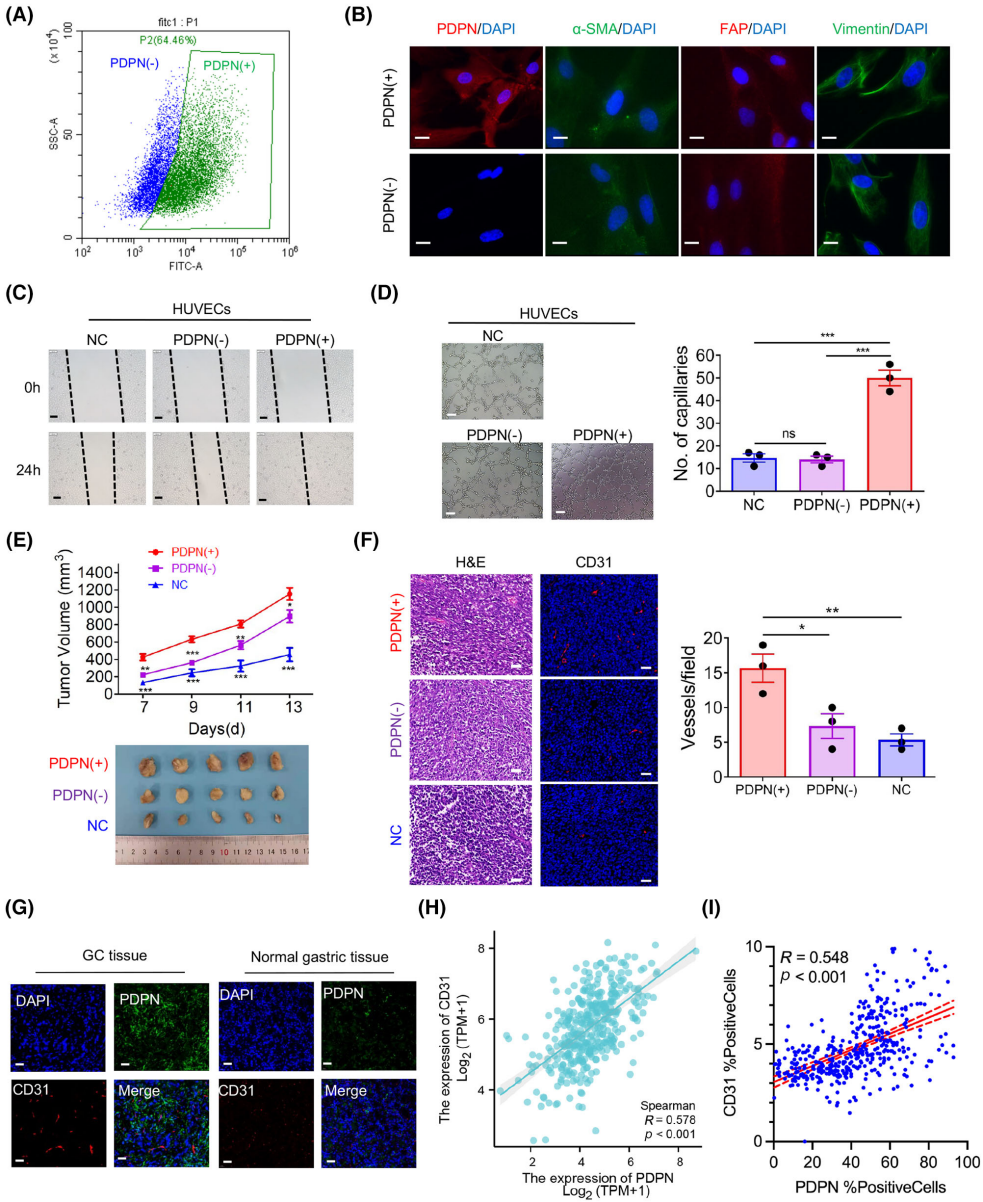
Podoplanin (PDPN)(+) cancer‐associated fibroblasts (CAFs) induce angiogenesis in vitro and in vivo. (A) PDPN(+) CAFs were sorted by fluorescence‐activated cell sorting (FACS) after incubation with FITC‐conjugated anti‐human PDPN antibody. (B) Immunofluorescent staining showing the expression PDPN, alpha‐smooth muscle actin (α‐SMA), and fibroblast activation protein (FAP) expression in PDPN(+) CAFs and PDPN(−) CAFs (scale bar = 25 µm). (C, D) Wound healing assay (C) and tube formation assay (C) performed on human umbilical vein endothelial cells (HUVECs) cultured in conditioned medium (CM) derived from PDPN(+) CAFs and PDPN(−) CAFs (scale bar = 100 µm; ***p < 0.001). (E) Tumor growth curve (upper) and representative tumor size at day 13th (lower) for each group of xenograft mice (*n* = 5 per group) are shown (*p < 0.05, ***p < 0.001). (F) Representative hematoxylin and eosin (H&E) staining and immunofluorescent staining images (left), along with quantitative results (right), showing CD31 expression in harvested xenograft tumors (scale bar = 40 µm; *p < 0.05). (G) Representative immunofluorescent staining for DAPI, PDPN and CD31 in clinical gastric cancer (GC) and normal gastric tissues (scale bar = 40 µm). (H) Correlation of PDPN and CD31 mRNA expression in tumor tissue samples of 407 GC patients in the TCGA dataset. (I) Correlation of PDPN and CD31 mRNA expression, detected by immunohistochemistry (IHC), in tumor tissue samples of 400 GC patients in the in‐house dataset.

Given that PDPN(+) CAFs indicate an activated state within the TME and that elevated PDPN levels correlate with tumor angiogenesis in GC, we hypothesize that PDPN‐expressing CAFs represent a subset that promotes angiogenesis. To test our hypothesis, we isolated PDPN(−) CAFs and PDPN(+) CAFs using fluorescence‐activated cell sorting (FACS; Figure 3A). The purity of these sorted populations was confirmed through immunofluorescence staining for PDPN and fibroblast markers, alpha‐smooth muscle actin (α‐SMA) and FAP (Figure 3B). To evaluate the effect of PDPN(+) CAFs on the angiogenic behavior of vascular ECs, we employed a coculture system with HUVECs and conditioned medium (CM) derived from either PDPN(+) CAFs or PDPN(−) CAFs. The findings revealed that PDPN(+) CAFs significantly improved the migratory and tube‐forming capabilities of HUVECs compared to PDPN(−) CAFs (Figure 3C,D).

To further investigate the proangiogenic and tumor‐promoting effects of PDPN(+) CAFs in vivo, we inoculated MKN45 cells subcutaneously into mice, either alone or in combination with PDPN(+) CAFs or PDPN(−) CAFs. Thirteen days later, the mice were euthanized, and it was observed that MKN45 cells formed larger tumors when coinjected with PDPN(+) CAFs compared to PDPN(−) CAFs or MKN45 cells alone (Figure 3E). Moreover, immunofluorescence staining for CD31 revealed that vessel density within tumors was significantly increased in the PDPN(+) CAFs group compared to the other two groups (Figure 3F). Consistent with these findings, immunofluorescence staining of tumor and normal gastric tissues from GC patients showed an abundance of CD31 in areas with PDPN(+) CAFs (Figure 3G). In a cohort of 407 GC patients from the TCGA dataset, we found a significant positive correlation between PDPN mRNA expression levels and CD31 expression (*r* = 0.578, *p* < 0.001; Figure 3H). Additionally, in an in‐house GC cohort, immunohistochemistry (IHC) revealed a strong positive correlation between PDPN and CD31 protein expression (*r* = 0.548, *p* < 0.001; Figures 3I and 5A). Collectively these data underscore that PDPN(+) CAFs are a prominent proangiogenic factor in GC, playing a critical role in promoting tumor growth and vascularization.

### 2.4 PDPN(+) CAFs induce angiogenesis by secreting CCL2

**FIGURE 4:**
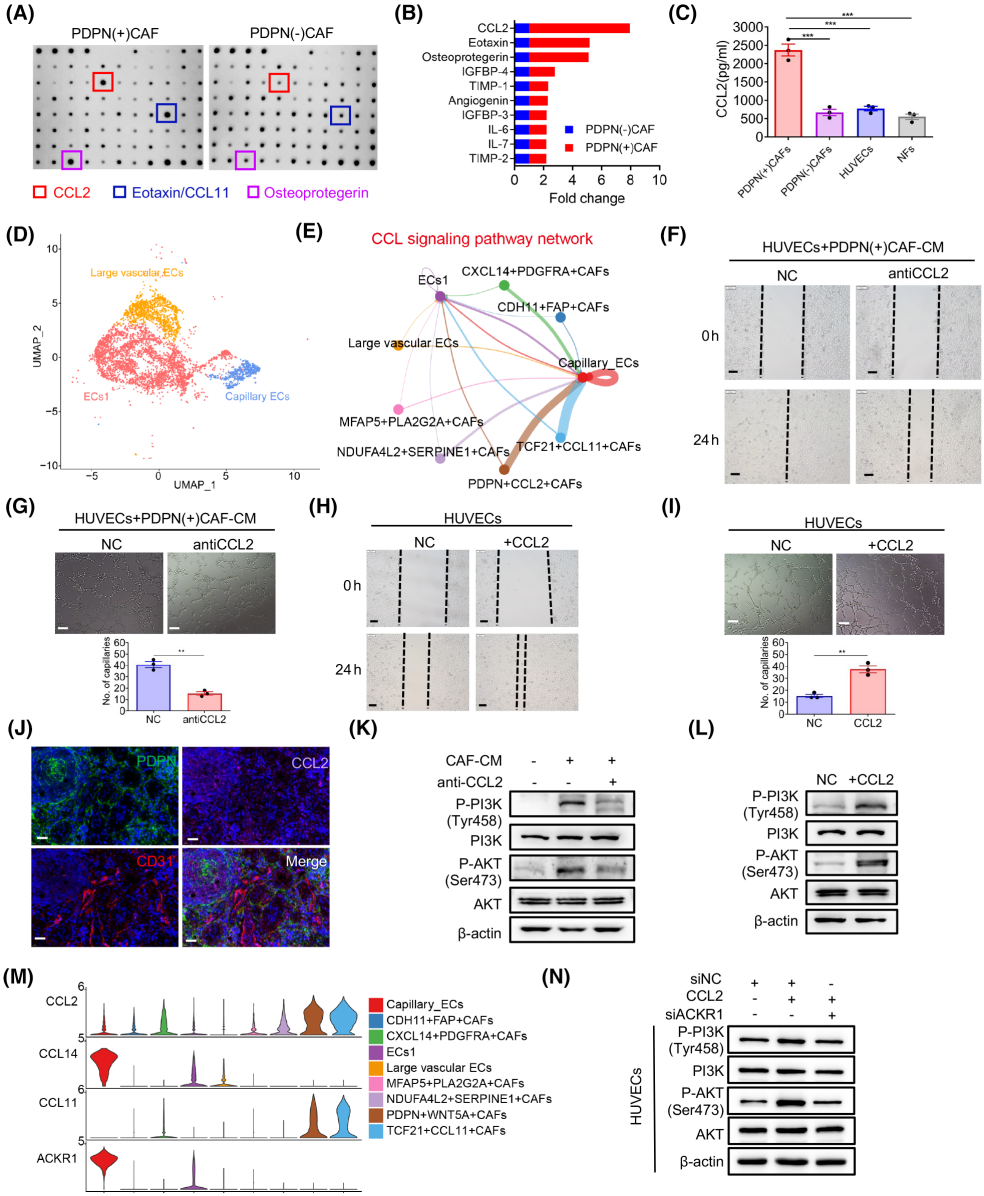
Podoplanin (PDPN)(+) cancer‐associated fibroblasts (CAFs) induce angiogenesis by secreting chemokine (CC‐motif) ligand 2 (CCL2). (A) Cytokine profiles produced by conditioned medium (CM) of PDPN(+) CAFs and PDPN(−) CAFs were examined by RayBio Human Cytokine Antibody Array. The red, blue, and purple frame represents the most significantly overexpressed cytokines. (B) Top 10 cytokines significantly upregulated in CM of PDPN(+) CAFs compared to PDPN(−) CAFs (***p < 0.001). (C) Enzyme‐linked immunosorbent assay (ELISA) results showing the amount of soluble CCL2 produced by PDPN(+) CAFs, PDPN(−) CAFs, normal fibroblasts (NFs), and human umbilical vein endothelial cells (HUVECs). (D) Uniform manifold approximation and projection (UMAP) of endothelial cells representing three unique cell states, color‐coded by their corresponding cell lineage or subtype. Each dot in the UMAP represents a single cell. (E) Circle plots showing the cellular interactions of CAFs and endothelial cells (ECs) involved in the CCL signaling pathway network in gastric cancer (GC). CAFs and ECs were the core of the cellular interaction network (edge width represents the numbers of interactions and node size represents the abundance of cell populations). (F, G) Wound healing assay (F) and tube formation assay (G) on HUVECs cultured in CM of PDPN(+) CAFs with or without a neutralizing antibody against CCL2 (anti‐CCL2). Scale bar = 100 µm; **p < 0.01. (H, I) Wound healing assay (H) and tube formation assay (I) on HUVECs treated with or without CCL2 (scale bar = 100 µm; **p < 0.01). (J) Representative immunofluorescent staining for PDPN, CCL2, and CD31 in clinical GC sample (scale bar = 40 µm). (K) Immunoblot analysis of p‐PI3K, PI3K, p‐AKT, AKT expression in HUVECs treated with CM from PDPN(+) CAFs and CCL2 neutralizing antibody (anti‐CCL2). (L) Immunoblot analysis of p‐PI3K, PI3K, p‐AKT, AKT expression in HUVECs treated with CCL2. (M) Violin plots showing the expression of genes involved in the CCL signaling pathway. CCL2 and CCL11 are primarily expressed in fibroblast subsets, while their potential receptor ACKR1, is predominantly expressed in endothelial cells. (N) Immunoblot analysis of p‐PI3K, PI3K, p‐AKT, AKT expression in HUVECs with indicated treatment.

To gain deeper insight into the mechanisms underlying PDPN(+) CAFs‐mediated angiogenesis, we performed antibody microarrays to compare the cytokine profiles produced by PDPN(+) CAFs and PDPN(−) CAFs (Figure 4A). Among the identified cytokines, CCL2 emerged as the most significantly differentially expressed cytokine (Figure 4B). We additionally confirmed that CCL2 concentrations were significantly elevated in the supernatant of PDPN(+) CAFs when compared to PDPN(−) CAFs, NFs, and HUVECs, as determined by enzyme‐linked immunosorbent assay (ELISA; Figure 4C). To identify the specific EC subtypes affected by PDPN(+) CAFs, we analyzed 6992 ECs and characterized three distinct subclusters based on unique gene expression profiles: ECs1, large vascular ECs, and capillary ECs (Figure 4D and S2A). Constructing the cellular interaction network (Figure S2B) and calculating the interaction weight/strength (Figure S2C), we found that the CCL signaling pathway network was the primary interaction network between PDPN(+) CAFs and capillary ECs (Figure 4E). Consistent with this finding, treating the CM of PDPN(+) CAFs with a neutralizing antibody against CCL2 significantly reduced the migration and tube‐forming abilities of HUVECs (Figure 4F,G). Moreover, inhibiting CCL2 with small interfering RNA (siRNA; Figure S2D) in PDPN(+) CAFs also substantially abrogated their proangiogenic effects on HUVECs (Figure S2E,F). Furthermore, recombinant CCL2 alone was sufficient to stimulate the migratory potential and tube‐forming ability of HUVECs (Figure 4H,I). In agreement, immunofluorescent staining of GC patient tumor samples revealed that CCL2 was abundantly expressed in PDPN(+) CAFs (Figure 4J).

The scRNA‐seq data also indicated that CCL2 was predominantly expressed in CAFs and vascular ECs, identified by PDPN and CD31, respectively (Figures 1L and S2G). Furthermore, in our in‐house IHC cohort, the expression of CCL2 protein exhibited a slight positive correlation with PDPN (*r* = 0.126, *p* = 0.012) and CD31 (*r* = 0.123, *p* = 0.014; Figures S2H and 5A). In the TCGA dataset of 407 GC patients, CCL2 mRNA expression was significantly correlated with PDPN (*r* = 0.504, *p* < 0.001) and CD31 (*r* = 0.515, *p* < 0.001) expression (Figure S2I). Collectively, these findings suggest that PDPN(+) CAFs secrete CCL2 to promote angiogenesis.

**FIGURE 5:**
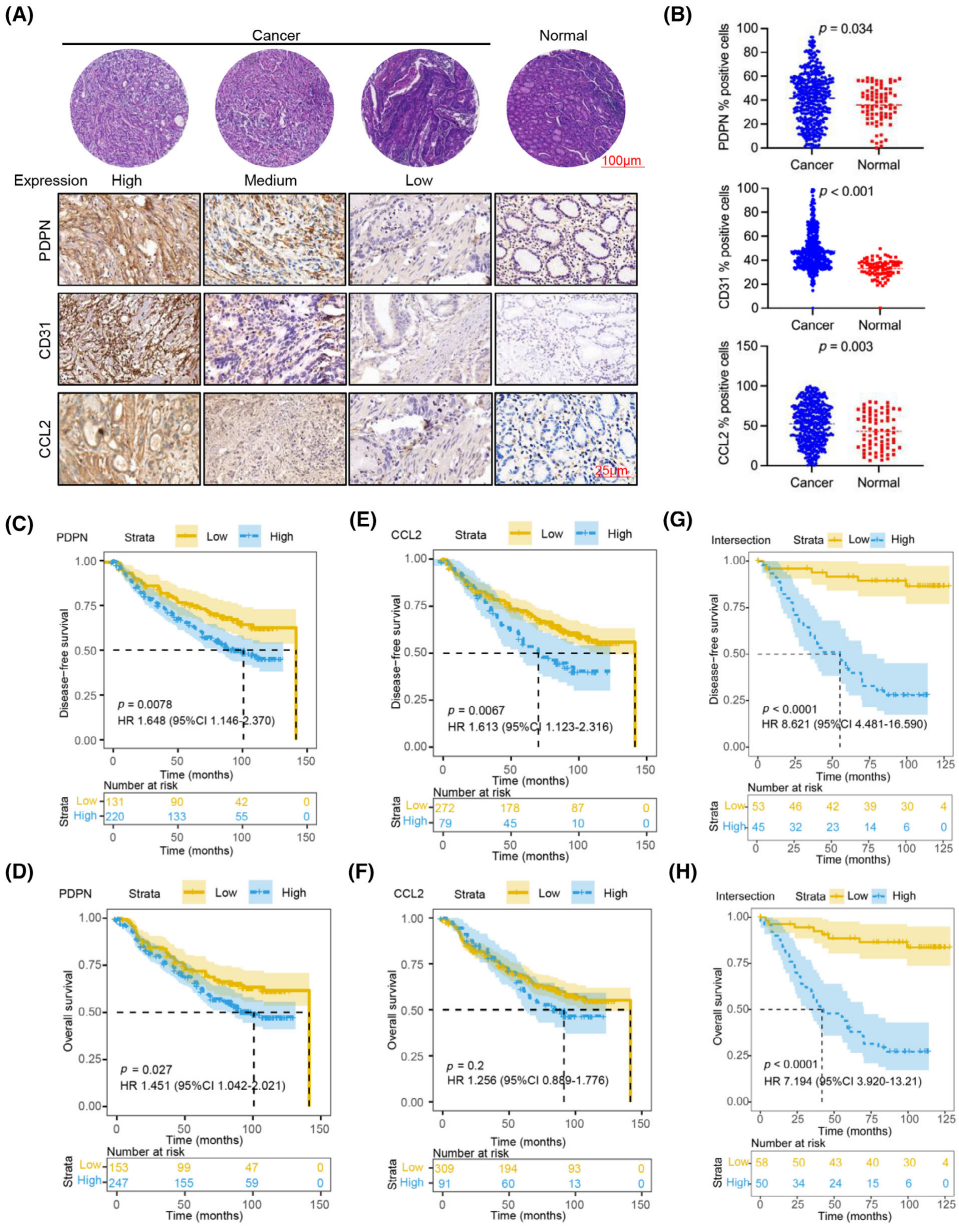
High expression of podoplanin (PDPN), CD31 and chemokine (CC‐motif) ligand 2 (CCL2) in gastric cancer (GC) is associated with poor prognosis. (A) Representative hematoxylin and eosin (H&E) and immunohistochemical images showing PDPN, CD31, and CCL2 protein expression in GC and normal gastric mucus; (B) Semiquantitative analysis of PDPN, CD31, and CCL2 protein expression between GC and normal gastric mucosa; (C–H) Kaplan–Meier survival curves with log‐rank tests depicting disease‐free survival (C, E, G) and overall survival (D, F, H) of GC patients (*n* = 400) based on the expression levels of PDPN, CCL2, and intersection of all three proteins in cancer tissues in the in‐house cohort.

### 2.5 PDPN(+) CAFs increase CCL2 secretion, activating PI3K/AKT signaling via ACKR1 in endothelial cells

Since CCL2 is known to activate the PI3K/AKT signaling pathway in various tumors,^23^ we hypothesized that CCL2 derived from CAFs stimulates ECs via the PI3K/AKT pathway. As shown in Figure 4K, western blotting confirmed that the phosphorylation of PI3K (p‐PI3K) and AKT (p‐AKT) was elevated in HUVECs treated with PDPN(+) CAFs compared to the untreated group. However, treatment of the CM from PDPN(+) CAFs with a neutralizing antibody against CCL2 significantly decreased p‐PI3K and p‐AKT levels in HUVECs. Additionally, recombinant CCL2 alone activated the PI3K/AKT signaling in HUVECs (Figure 4L). These findings suggest that CCL2 derived from PDPN(+) CAFs activates the PI3K/AKT pathway in ECs. To further investigate the role of the PI3K/AKT pathway in CAF‐mediated angiogenesis, we treated HUVECs with two PI3K/AKT inhibitors, perifosine and LY294002, which significantly inhibited CCL2‐induced migration and tube formation in HUVECs (Figure S2J,K). Immunochemistry of tumor tissues harvested earlier also confirmed that the group with PDPN(+) CAFs expressed higher levels of PDPN, CCL2, and p‐AKT in the tumor stroma compared to the PDPN(−) CAFs group and NC group (Figure S3A). These results indicate that CCL2 derived from PDPN(+) CAFs mediates angiogenesis by activating the PI3K/AKT signaling pathway in ECs.

To identify the key mediators of PDPN(+) CAFs and ECs interaction in GC, we investigated the mechanisms of cell–cell communication between CAFs and ECs. Utilizing the R package “CellChat,” we assessed the potential crosstalk based on the expression of ligand‐receptor pairs. Our analysis indicated that CCL2 from PDPN(+) CAFs may directly interact with ECs through the ACKR1 receptor (Figures 4M and S3B). In the TCGA GC dataset, the mRNA expression level of CCL2 was significantly positive correlated with ACKR1 (*r* = 0.472, *p* < 0.001) expression (Figure S3C). Moreover, ACKR1 expression on HUVECs significantly increased following CCL2 treatment, which was reversed by the anti‐CCL2 antibody (Figure S3D). Furthermore, the knockdown of ACKR1 partially inhibited CCL2‐induced activation of the PI3K/AKT signaling pathway in HUVECs (Figure 4N). Collectively, these findings suggest that CCL2 produced by PDPN(+) CAFs activates ACKR1+ ECs, thereby promoting angiogenesis via the PI3K/AKT signaling pathway.

### 2.6 High expression of PDPN, CD31, and CCL2 in GC is associated with poor prognosis

To clinically evaluate the expression levels of PDPN, CD31, and CCL2 in GC samples, we assessed protein levels in 400 tumor and 73 normal gastric tissue samples (Figure 5A). Our analysis revealed that PDPN, CD31, and CCL2 were all highly expressed in GC tissues compared to normal tissues (Figure 5B). We classified all samples into high and low protein expression groups by calculating the Youden index for each protein and then analyzed their correlation with patients’ survival times. Patients with high PDPN protein levels exhibited significantly lower disease‐free survival (DFS; *p* = 0.0078; Figure 5C) and overall survival (OS; *p* = 0.027; Figure 5D) rates. Similarly, high CD31 protein expression was associated with OS (*p* < 0.0001; Figure S4A) and decreased DFS (*p* < 0.0001; Figure S4B) rates. Additionally, GC patients with elevated CCL2 protein expression showed lower DFS rates (*p* = 0.0067; Figure 5E), although the correlation with OS was not as strong (*p* = 0.2; Figure 5F). Notably, when we reclassified GC patients based on the combined expression levels of PDPN, CD31, and CCL2, those with high levels of all three proteins had significantly worse DFS (*p* < 0.0001; Figure 5G) and OS rates (*p* < 0.0001; Figure 5H) compared to those with low levels. These findings suggest that the combined high expression of PDPN, CD31, and CCL2 in GC tissues could serve as a novel and effective prognostic indicator for patients with GC.

### 2.7 PDPN regulates NF‐κB activity in CAFs through AKT/IKK signaling, leading to transcriptional induction of CCL2 secretion

**FIGURE 6:**
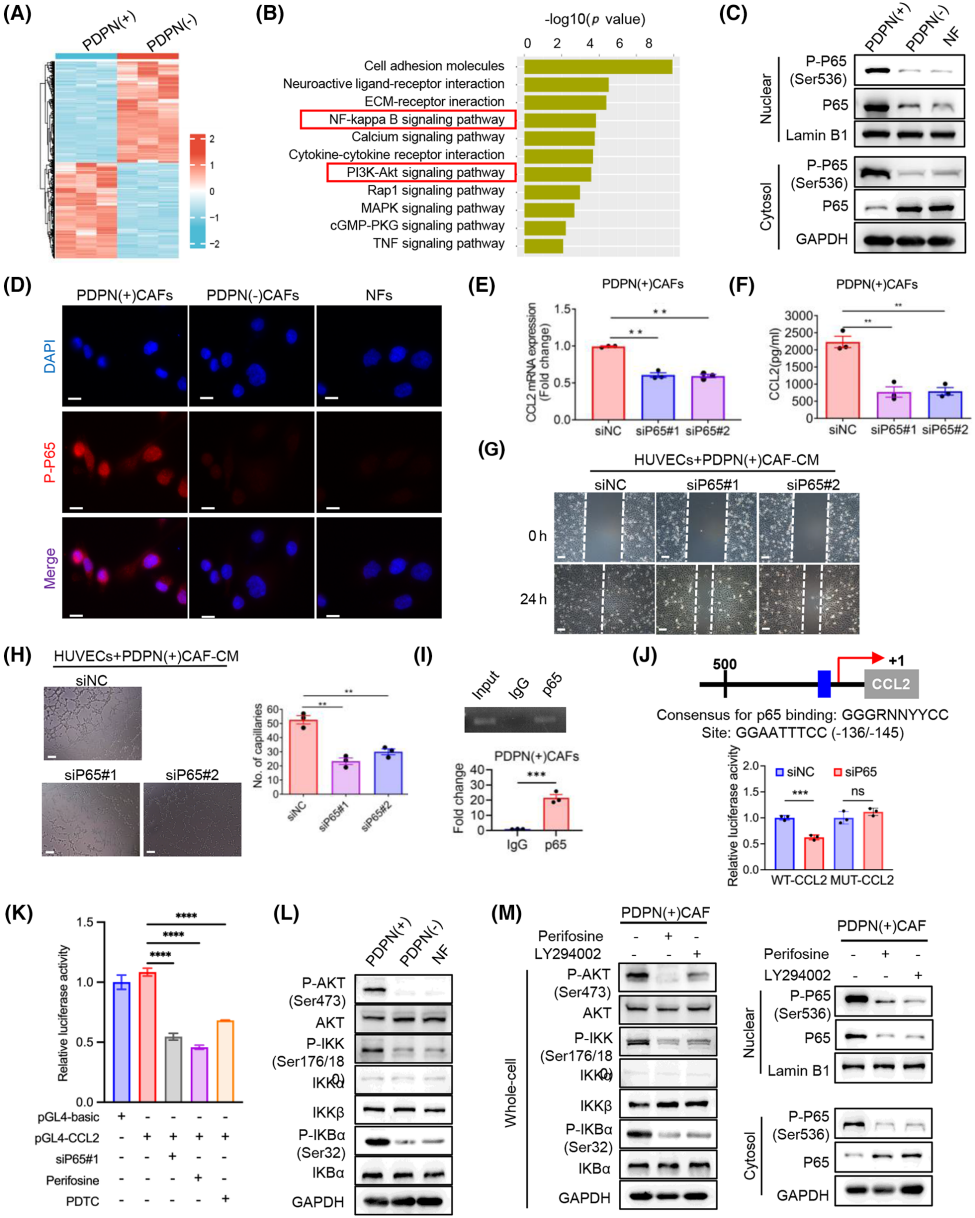
Podoplanin (PDPN) regulates NF‐κB activity in cancer‐associated fibroblasts (CAFs) through AKT/IKK signaling, leading to chemokine (CC‐motif) ligand 2 (CCL2) secretion. (A) Heatmap representing significantly dysregulated genes from RNA‐seq analysis of PDPN(+) CAFs and PDPN(−) CAFs (*n* = 3). (B) Pathway analysis of differentially expressed genes enriched in PDPN(+) CAFs compared to PDPN(−) CAFs. (C) Immunoblot analysis of nuclear and cytoplasmic p‐P65 and P65 protein expression in PDPN(+) CAFs, PDPN(−) CAFs and normal fibroblasts (NFs). (D) Representative immunofluorescent staining of p‐P65 in PDPN(+) CAFs, PDPN(−) CAFs, and NFs (scale bar = 25 µm). (E) Quantitative reverse transcription polymerase chain reaction (qRT‐PCR) results showing the differential mRNA expression levels of CCL2 in PDPN(+) CAFs transfected with P65 siRNA or control siRNA (*n* = 3; **p < 0.01). (F) Enzyme‐linked immunosorbent assay (ELISA) results showing the amount of soluble CCL2 in conditioned medium (CM) from PDPN(+) CAFs transfected with P65 siRNA or control siRNA (**p < 0.01). (G, H) Wound healing assay (G) and tube formation assay (H) on human umbilical vein endothelial cells (HUVECs) treated with CM from PDPN(+) CAFs transfected with P65 siRNA or control siRNA (scale bar = 100 µm; **p < 0.01). (I) Chromatin immunoprecipitation (ChIP) assay was performed to verify P65 binding to the CCL2 promoter. CCL2 promoter segments were quantified using qRT‐PCR, with results normalized against IgG. Data are presented as the mean ± SD from three independent experiments is presented (***p < 0.001). (J) Top: Schematic representation of the CCL2 reporter construct. The consensus P65 binding sequences are marked as with blue box. The consensus sequence and the putative P65 binding site sequences are shown. Bottom: Effects of ectopic expression of P65 siRNA on wild type (WT) and mutant (MUT) CCL2 promoter reporter activity (**p < 0.01). (K) Effects of ectopic expression of P65 siRNA (siP65#1), AKT inhibitors perifosine (10 µmol/L), NF‐κB inhibitor pyrrolidine dithiocarbamate (PDTC, 0.05 µmol/L) on CCL2 promoter reporter activity (**p < 0.01). (L) Immunoblot analysis of AKT/IKK pathway in PDPN(+) CAFs, PDPN(−) CAFs and NFs. (M) Immunoblot analysis of AKT/IKK/NF‐κB pathway and nuclear/cytoplasmic distribution of P65 in PDPN(+) CAFs treated with or without perifosine (10 µmol/L) and LY294002 (20 µmol/L) for 24 h.

To explore the key signaling pathways involved in the regulation of CCL2 production by PDPN(+) CAFs, we performed a transcriptome analysis comparing PDPN(+) and PDPN(−) CAFs (Figure 6A). Kyoto Encyclopedia of Genes and Genomes (KEGG) enrichment analysis revealed a group of genes linked to the AKT and NF‐κB signaling pathways that were upregulated in PDPN(+) CAFs relative to PDPN(−) CAFs (Figure 6B). CCL2 is known to be a target gene of the NF‐κB pathway.^26^ Our findings demonstrated that the nuclear levels of phosphorylated P65 (p‐P65) and the accumulation of P65 in the nucleus were significantly higher in PDPN(+) CAFs than in PDPN(−) CAFs and NFs, while there was no significant difference in the whole‐cell levels of total P65 expression among the three groups (Figures 6C,D, and S4C,D).

To confirm whether NF‐κB is essential for PDPN(+) CAFs‐induced CCL2 secretion and angiogenesis, we knocked down P65 expression in PDPN(+) CAFs using siRNAs (Figure S4E). The results demonstrated that P65 suppression significantly reduced both the mRNA levels and secretion of CCL2 from PDPN(+) CAFs (Figure 6E,F), and diminished the migratory potential as well as tube‐forming ability of HUVECs (Figure 6G,H). Additionally, treating PDPN(+) CAFs with the NF‐κB inhibitor Bay117082 also decreased CCL2 production and its effects on HUVECs (Figure S4E‐G). Chromatin immunoprecipitation (ChIP) analysis revealed a P65 (encoded by RELA) binding region (−136/−145) on the promoter of CCL2 (Figure 6I). Inhibition of NF‐κB suppressed the activity of the CCL2 promoter reporter, but had no significant effect at the presence of mutation in the binding site (Figure 6J,K). These results demonstrate that NF‐κB activation in PDPN(+) CAFs transcriptionally activates CCL2 secretion, enhancing their proangiogenic function.

Given that NF‐κB activation is often mediated through the AKT/IKK pathway, we hypothesized that the AKT/IKK/NF‐κB signaling axis sustains the phenotypes and functions of PDPN(+) CAFs. Western blot analysis demonstrated a notable elevation in the levels of phosphorylated AKT (p‐AKT), phosphorylated IKK (p‐IKK), and phosphorylated IκB‐α (p‐IκB‐α) in PDPN(+) CAFs compared to both PDPN(−) CAFs and NFs, while the total levels of AKT, IKK, and IκB‐α remained unchanged (Figure 6L). To further validate that the AKT/IKK pathway modulates NF‐κB activation in PDPN(+) CAFs, we treated these CAFs with two AKT inhibitors, perifosine and LY294002. As anticipated, both inhibitors significantly suppressed the activation of AKT/IKK signaling and subsequently blocked the nuclear translocation of P65 and p‐P65 in PDPN(+) CAFs (Figure 6M). These results demonstrate that NF‐κB activation in PDPN(+) CAFs was mediated by the AKT/IKK pathway, driving the secretion of CCL2 and promoting angiogenesis.

### 2.8 Targeting PDPN(+) CAFs restrains angiogenesis via inhibiting AKT/NF‐κB signaling

**FIGURE 7:**
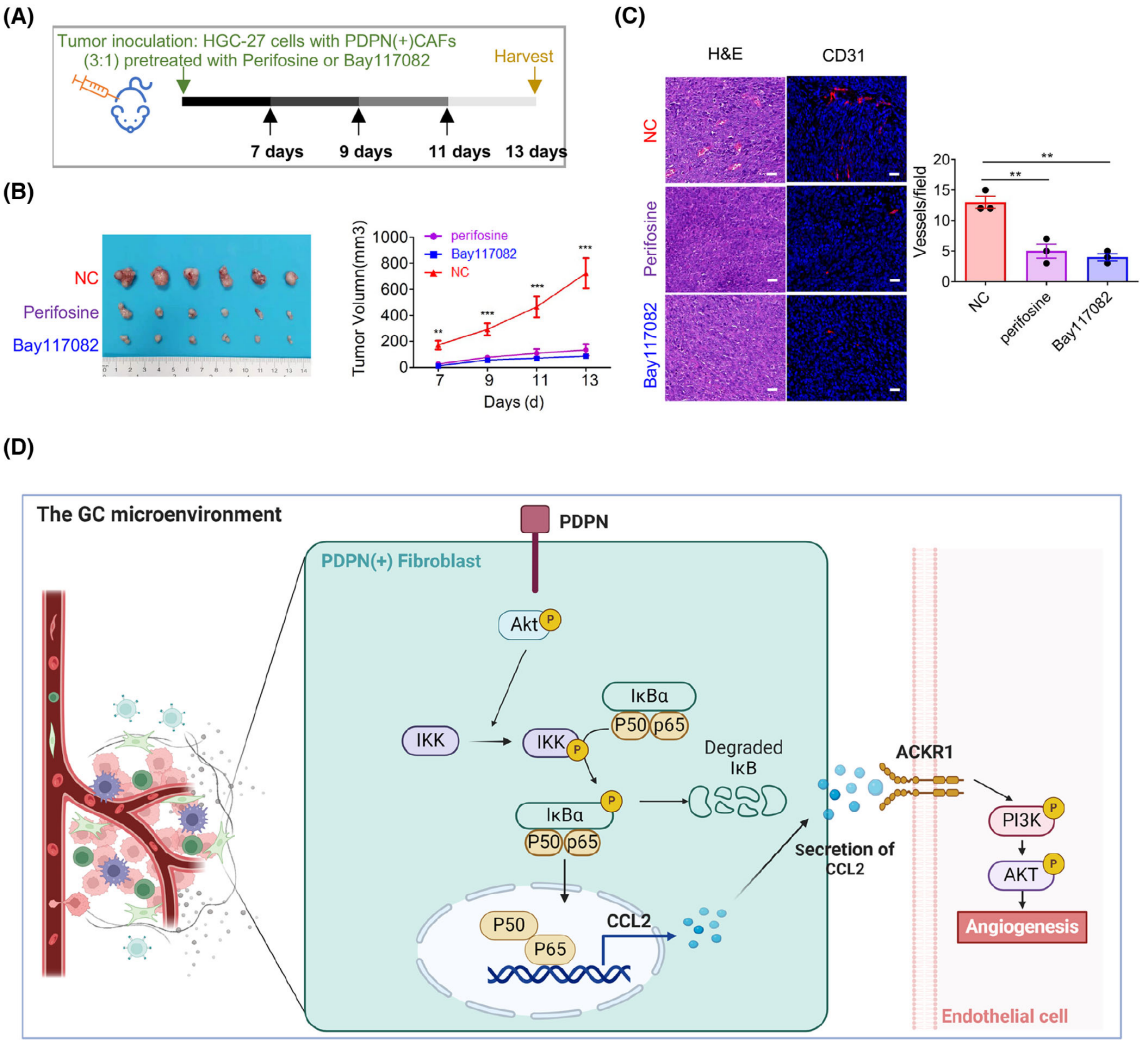
Targeting podoplanin (PDPN)(+) cancer‐associated fibroblasts (CAFs) restrains angiogenesis via inhibiting AKT/NF‐κB signaling. (A) Schematic diagram of in vivo animal experiment. (B) Tumor image (left) and tumor growth curve (right) of xenografted mice inoculated with HGC27 cells and PDPN(+) CAFs pretreated with or without perifosine (10 µmol/L) and Bay117082 (10 µmol/L; ***p < 0.001). (C) Representative images of hematoxylin and eosin (H&E) and immunofluorescent staining for CD31 as indicated in the xenografted tumors (scale bar = 40 µm; *p < 0.05). (D) Schematic view of the proposed mechanism. A schematic diagram illustrating the proposed mechanism by which PDPN(+) CAFs‐derived chemokine (CC‐motif) ligand 2 (CCL2) promotes angiogenesis in gastric cancer (GC). The Akt/NF‐κB pathway was significantly activated in PDPN(+) CAFs. P65 directly bind to the CCL2 promoter, thereby increasing the CCL2 transcription and secretion in CAFs; CCL2, which was transcriptional activated by P65 in PDPN(+) CAFs, sustains tumor angiogenesis by interacting with ACKR1 and activating PI3K/AKT signaling in endothelial cells. The scheme was drawn by biorender (https://biorender.com/).

To further investigate the therapeutic potential of targeting the AKT/IKK/NF‐κB signaling pathway in PDPN(+) CAFs, we pretreated PDPN(+) CAFs with the AKT inhibitor perifosine and the NF‐κB inhibitor Bay117082, then subcutaneously inoculated these cells into nude mice alongside with HGC‐27 cells. Thirteen days postinjection, the tumors were collected for analysis (Figure 7A). As anticipated, treating PDPN(+) CAFs with either perifosine or Bay117082 significantly inhibited the tumor growth (Figure 7B). Immunofluorescent staining for CD31 revealed that blocking the AKT/IKK/NF‐κB signaling pathway in PDPN(+) CAFs resulted in a substantial decrease in tumor vessel density (Figure 7C). Collectively, our data suggest that the AKT/IKK/NF‐κB pathway in PDPN(+) CAFs regulates CCL2 secretion, which subsequently stimulates angiogenesis and tumor growth. Targeting PDPN(+) CAFs may represent an effective therapeutic strategy for GC treatment (Figure 7D).

## 3 DISCUSSION

In recent years, the significant role of CAFs in promoting growth and metastasis by inducing angiogenesis has been increasingly recognized in various malignancies, including breast cancer,^27^ melanoma,^28^ and colorectal cancer.^29^ However, the complex process of CAF involvement in GC angiogenesis remains poorly understood. Recent extensive research has demonstrated that CAFs are heterogeneous,^8^, ^9^ not only in their diverse sources but also in the existence of different molecular and functional phenotypes within the TME. This study aims to uncover specific subsets of CAFs that play a role in angiogenesis, examining their importance in the interactions between the TME and cancer cells, along with their underlying molecular biological mechanisms. Such insights will undoubtedly enhance our understanding of tumor development and inform targeted therapeutic strategies. Establishing the relevance of our experimental findings to human malignancy, PDPN(+) CAFs are one of the major constituents of activated CAFs (anCAFs) in human GC stroma. The overexpression of the PDPN/CCL2 axis is a classifier of disease outcome in GC. Mechanically, we found that PDPN activates transcription factor P65 of fibroblasts to upregulate CCL2 expression, and PDPN(+) CAFs secreted CCL2 maintains PI3K/AKT signaling activation in ECs via recognizing ACKR1 receptor. Moreover, targeting PDPN(+) CAFs restores angiogenesis via inhibiting AKT/NF‐κB signaling.

Here, we have discovered that CAFs play a crucial role in tumor angiogenesis of GC. CAFs can recruit and stimulate ECs to form vessels and buds. By conducting a transcriptional gene analysis using NFs and CAFs, we found that PDPN, a membrane protein previously reported to have oncogenic effects in CAFs^30^, ^31^, ^32^ is significantly enriched in some CAFs with potential angiogenic functions, defining a subset of human GC's angiogenic CAFs (anCAFs).More importantly, the use of PDPN surface markers enables cell classification in live cells, which is crucial for revealing the function and mechanism of this subset of CAFs. Recent studies have utilized single‐cell RNA sequencing approaches to characterize the CAFs subtypes involved in vascularization. In endometrial cancer, a spatially separated vascular CAFs subgroup characterized by high levels of MYH11, ESAM, MCAM, and EPAS1 was a poor prognostic factor for patients with endometrial cancer.^33^ In intrahepatic cholangiocarcinoma, a population of CD146‐positive vascular CAFs was identified as the majority CAFs subtype, participating in tumor progression partly by upregulating microvasculature signatures.^34^ Although these vascular CAFs do not exhibit high expression of PDPN, both share functional similarities with the subpopulations of anCAFs that we have identified. However, in breast cancer, it has been reported that PDPN(+) CAFs can diverge into protumorigenic CAFs during tumor progression,^31^ or promote resistance to trastuzumab in HER2+ cancer by secreting immunosuppressive factors.^30^ This suggests the complexity of the heterogeneity of CAFs in human cancers. It indicates that surface molecular markers expressed in CAFs subpopulations with the same functional phenotype may be different in different tumors, and CAFs subpopulations with the same surface molecular markers may also have different functional manifestations in different tumors.

Although our previously reported that PDPN(+) CAFs promoted GC cells metastasis by secreting POSTN,^18^ the present study shows that cytokine CCL2, in addition to well‐known vascularization regulators interleukin‐6 (IL‐6), IL‐8, and VEGFA, is a novel proangiogenic factor regulated by PDPN in gastric stromal CAFs. Previously, CCL2 was found to play a role in macrophage recruitment and activation, thus involving in angiogenesis in GC.^35^, ^36^ Recently, a study proved that CCL2 is the cytokine with angiogenic biological activities toward mast cells. CCL2 secreted from mast cells exhibits proinflammatory and chemotactic properties and is associated with tumor angiogenesis in lung cancer.^37^ Neutrophils‐derived CCL2 also has the capacity to promote proliferation and migration of GC cells.^38^ Here, we confirm that CCL2 secreted from PDPN(+) CAFs acts as a powerful mediator between CAFs and ECs to facilitate tumor angiogenesis. The activation of the AKT/IKK/NF‐κB pathway in stromal CAFs enhances CCL2 secretion, but not the known proangiogenic factors such as VEGF, Stromal Cell Derived Factor 2 (SDF), FGFs, or PDGF, to stimulate the PI3K/AKT signaling activation and promote tubule formation and sprouting of HUVECs.

While membrane proteins are often highlighted for their involvement in conveying signals across the cell membrane, other membrane proteins contribute significantly to cell structure, substance transport, and cell recognition. A notable example can be observed in the research conducted by Su et al.,^39^ their study revealed that the activation of the NF‐κB in CD10(+)GPR77(+) CAFs led to the secretion of elevated levels of IL‐6 and IL‐8. Interestingly, the study found that silencing GPR77—rather than CD10—resulted in a marked reduction in the secretion of IL‐6 and IL‐8, along with the inhibition of NF‐κB activity. This finding underscores the significance of specific membrane proteins in mediating cellular responses and suggests that GPR77 operates as a crucial player in this signaling cascade. Such insights pave the way for further investigation into other potential signaling molecules or surface proteins, particularly within the context of PDPN(+) CAFs, which molecule may trigger the activation of the AKT signaling pathway. Despite these advancements, the precise intracellular signaling molecules or surface markers that drive AKT/IKK pathway in PDPN(+) CAFs remain unidentified. As such, this gap in knowledge identifies a critical area for future research endeavors. By elucidating the specific roles of various membrane proteins and their interactions, we may uncover novel therapeutic targets to mitigate GC progression and improve patient outcomes.

Despite the identification of a new subpopulation of GC‐related proangiogenic PDPN(+) CAFs and the mechanism by which PDPN(+) CAFs secrete CCL2 to activate the ACKR1/PI3K/AKT signaling in ECs, this study has several limitations. First, while PDPN is recognized as a classic CAF marker implicated in tumorigenesis,^20^, ^40^ further molecular biology analysis is required to understand how this membrane protein activates the PI3K/IKK/NF‐κB signaling pathway.^41^ Second, single‐cell sequencing data suggest that PDPN(+) CAFs may originate from TCF21^+^CCL11^+^ CAFs and CXCL14^+^PDGFRA^+^ CAFs subpopulations, but the evolution of molecular expression during this CAF transformation process remains unclear. Additionally, prospective collection of tissue samples from patients with antiangiogenic resistance for detecting PDPN(+) CAFs subgroups and angiogenesis is crucial for fully understanding the clinical value of PDPN(+) CAFs subpopulations.

The CCL2 produced by PDPN(+) CAFs activates the PI3K/AKT signal transduction pathway via recognizing the surface receptor ACKR1, which is the primary mechanism of EC activation. When using AKT inhibitors perifosine and LY294002, both HUVECs treated with CCL2 and tumor‐burdened mice injected with PDPN(+) CAFs and GC cells maintained their proangiogenic properties. This research not only provides a molecular definition of the CAF subset associated with angiogenesis in GC but also elucidates the key molecular interaction mechanism and signal pathways required to maintain the characteristics and functions of this specific matrix subtype. Overall, the activation of the AKT/NF‐κB signaling pathway in PDPN(+) CAFs promotes CCL2 secretion and enhances the ACKR1/PI3K/AKT signal transduction in ECs, thereby stimulating angiogenesis in GC. This study suggests that targeting the PDPN/CCL2 axis in CAFs may be an effective supplementary strategy to overcome resistance to anti‐VEGFR therapy in GC treatment.

## 4 CONCLUSION

Collectively, our results revealed that PDPN acts as a gatekeeper in angiogenesis, regulating mechanotransduction and Akt signaling in CAFs and ECs. This provides new insights into the complex interplay between signaling pathways and cellular processes in GC and suggests potential therapeutic strategies for this disease.

## 5 MATERIALS AND METHODS

### 5.1 Patients and samples

We obtained a series of 10 × 12 TMA containing gastric tissue samples from our hospital's Biobank. Each case included a repeat core to prevent sample loss. A total of 400 tumor and 73 adjacent normal tissue samples were interpretable in the TMA analysis. The study was approved by the Research Ethics Committee, and all patients provided informed consent (Ethical code: 050432‐4‐2108).

### 5.2 Cell lines

Human GC cell lines HGC27 and MKN45 were procured from Guangzhou Cellcook Biotech Co., Ltd. They were cultured in RPMI 1640 (Cat# C22400500BT, Gibco), with 10% fetal bovine serum (FBS, Cat# 10270‐106, Gibco) and 1% penicillin‐streptomycin (Cat# 15140122, Gibco). HUVECs were obtained from the China Center of Type Culture Collection (CCTCC, Wuhan, China) and cultured per the supplier's instructions.

### 5.3 Isolation of fibroblasts and preparation of conditioned medium

We extracted and cultured CAFs and NFs from human GC and normal tissues of patients who had surgery at our hospital, according to the previously outlined procedure.^42^ The supernatant was collected, centrifuged, and designated as CAFs‐CM.

### 5.4 Reagents and antibodies

Western blot antibodies include monoclonal mouse anti‐β‐actin (1:5000 dilution, Cat# AF7018, Affinity), PI3K (Cat# 4257, CST), P‐PI3K (Cat# 4228, CST), AKT (Cat# 4685, CST), P‐AKT (Cat# 4060, CST), IKKa (Cat# 11930, CST), IKKb (Cat# 8943, CST), p‐IKK (Cat# 2697, CST), p‐P65 (Cat# 3033, CST), P65 (Cat# 8242, CST), IκBa (Cat# 4814, CST), p‐IκBa (Cat# 2859, CST), GAPDH (Cat# 5174, CST), PDPN (Cat# sc‐376695, Santa Cruz) at 1:1000 dilution, and secondary antibody‐goat anti‐Rabbit and Mouse (Cat# M21003, Abmart). Other antibodies include CD31 (Cat# ab81289, Abcam), CCL2 neutralizing antibody (Cat# MAB679, R&D), human CCL2 (Cat# 300‐04‐5, PeproTech). Inhibitors include LY294002 (20 µM, Cat# S1105, Selleck) and perifosine (10 µM, Cat# HY‐50909, MCE) and Bay 11‐7082 (10 µM, Cat# S2913, Selleck).

### 5.5 Immunofluorescence staining for cells

We immunofluorescence‐stained cells according to the previously outlined procedure.^42^ Primary antibodies include PDPN (Cat# sc‐376695, Santa Cruz, dilution 1:100), FAP (Cat# 66562, CST, dilution 1:100), α‐SMA (Boster, dilution 1:100), and Vimentin (Cat# 5741, CST, dilution 1:100).

### 5.6 Fluorescence‐activated cell sorting

The cells were treated with a Fluorescein isothiocyanate (FITC)‐conjugated anti‐PDPN antibody (Cat# 337026, Biolegend), after which any excess antibody was eliminated through washing with phosphate‐buffered saline (PBS) supplemented with 0.5% FBS. Following rinsing with PBS, the sorting was conducted using FACS (#MoFloAstrios EQ).

### 5.7 Wound healing assay and tube formation assay

When HUVECs grew to over 90% confluence in a six‐well plate, a single scratch was made with a sterile 200 µL pipette tip. Detached cells were removed by PBS washing. The culture medium was then replaced with CAFs‐CM or RPMI‐1640 medium. Pictures were taken at 0 h and 24 h after scratch. For tube formation assay, Matrigel (Cat#354480, Corning) was added to each well of a 96‐well plate and HUVECs were seeded in a Matrigel‐coated well for 12 h, when HUVEC‐formed tubular networks were visualized by light microscopy.

### 5.8 RNA sequencing

We performed RNA sequencing according to the previously outlined procedure.^42^ All data are accessible in NODE (https://www.biosino.org/node) with the accession number OEP00005646 or through the URL: https://www.biosino.org/node/project/detail/OEP00005646.

### 5.9 Small interfering RNA transfection

We performed siRNA transfection according to the previously outlined procedure and harvested cells for further tests 48 h later.^18^ The sequence for siRNA targeting P65 and CCL2 was listed as follows: P65 siRNA1, GATTGAGGAGAAACGTAAA, P65 siRNA2, CCCACGAGCTTGTAGGAAA; CCL2 siRNA1, CCAAGCAGAAGTGGGTTCA, CCL2 siRNA2, CCAGTCACCTGCTGTTATA.

### 5.10 Enzyme‐linked immunosorbent assay

Cell culture solution was centrifuged and take the supernatant for ELISA. ELISA assays were run using CCL2 ELISA kits (Elabscience, E‐EL‐H6005) according to the manufacturer's instructions.

### 5.11 Human cytokine array and multiple immunofluorescences staining for tissues

We performed human cytokine array and multiple immunofluorescences staining for tissues according to the previously outlined procedure and the manufacturer's instructions.^18^

### 5.12 In vivo experiments

Male BALB/c‐nude mice (aged 5 weeks) were maintained in pathogen‐free conditions. We resuspended cancer cell lines (MKN45 or HGC27) alone with or without CAFs in serum‐free medium and injected them subcutaneously into flanks of the nude mice. From the seventh day, the length (*L*) and width (*W*) of the tumors were recorded. Tumor volume was determined using the formula: *V* = (*L* × *W* ^2^)/2. After a duration of 13 days, all mice were euthanized, and the tumors were surgically removed and measured. All animal procedures were approved by the ethics committee of the Experimental Animal Center at our hospital, Ethical code: FUSCC‐IACUC‐S2022‐0358.

### 5.13 Single‐cell RNA sequencing data analysis

To verify CAFs subsets in GC, we downloaded a previously published dataset from the GEO database (https://www.ncbi.nlm.nih.gov/geo/query/acc.cgi?acc=GSE183904). The data underwent initial filtering to retain genes expressed in at least three cells and cells expressing at least 200 genes, resulting in 24,492 genes and 158,641 cells. To further refine the dataset, potential doublets were removed using the DoubletFinder R package.^43^ We also calculated the proportions of mitochondrial and ribosomal RNA using the PercentageFeatureSet function. Stringent quality control measures were applied, retaining cells with gene expression between 200 and 4000 genes, mitochondrial content below 20%, and unique molecular identifier (UMI) counts under 20,000. After these filters, 139,322 cells were retained for further analysis.

The data from the 40 samples were then log‐normalized. We identified variable features by selecting the top 2000 highly variable genes using the FindVariableFeatures function. All genes were subsequently scaled using the ScaleData function. Dimensionality reduction was performed on the top 2000 highly variable genes using the RunPCA function. All functions, including NormalizeData, FindVariableFeatures, ScaleData, and RunPCA, were executed with default parameters. Data integration was performed using the Harmony algorithm.^44^ To construct a neighborhood graph, we used the FindNeighbors function, followed by uniform manifold approximation and projection (UMAP) dimensionality reduction with the RunUMAP function, specifying a dimensionality value of 40 and a resolution of 0.2, while other parameters were left at their defaults. Finally, cells were annotated according to cell markers from the GSE183904 source article and commonly recognized single‐cell annotation markers.

### 5.14 Pseudotime analysis

For pseudotime trajectory analysis, we employed the “DDRTree” algorithm within Monocle2^24^ (packageVersion = “2.22.0”) to infer the developmental trajectories of fibroblasts. We started by selecting the genes with the most significant variation across cells, as these are likely to drive changes in cell states. The highly variable genes identified during the preprocessing steps were utilized for this purpose. Cells were ordered in pseudotime based on gene expression dynamics to uncover potential lineage relationships. The pseudotime trajectories were visualized using Monocle2's plotting functions, and gene expression patterns were examined using “plot_genes_branched_heatmap” and “plot_multiple_branches_pseudotime.”

### 5.15 Cell–cell communication analysis

For analyzing fibroblast‐EC communications, we utilize the CellChat R package.^45^ This tool enables us to systematically explore and visualize detailed communication networks between these cell types based on single‐cell RNA sequencing data. We use the internal functions “netVisual_Chord_gene” to generate chord diagrams that illustrate cell–cell communication through specific ligands and receptors, and “plotGeneExpression” to create violin plots showing the expression levels of genes involved in the “CCL signaling” pathways between fibroblasts and ECs.

### 5.16 Statistical analysis

All statistical analyses were conducted using GraphPad Prism software. The results are expressed as means ± standard error of the mean (SEM). Comparisons between the two groups were made using an unpaired Student's *t*‐test. To assess OS and DFS differences between the groups, the Kaplan–Meier method with log‐rank test was employed. A *p* value of less than 0.05 was considered statistically significant.

## AUTHOR CONTRIBUTIONS

Mi‐die Xu, Zhenxiong Zhao, Yanqiu Zhang, and Hui Sun conceived the study, performed the literature search and bioinformatics analysis, and prepared the figures. Jinjia Chang, Xin Wang, Cong Tan, Shujuan Ni, Weiwei Weng, Meng Zhang, Dan Huang, Lei Wang, and Xu Wang helped with data collection, analysis, and interpretation. Mi‐die Xu, Wenchao Gu, and Weiqi Sheng wrote and revised the manuscript. All the authors read and approved the final manuscript.

## CONFLICT OF INTEREST STATEMENT

The authors declare no conflicts of interest.

## ETHICS STATEMENT

The study was approved by the Research Ethics Committee, and all patients provided informed consent (Ethical code: 050432‐4‐2108). All animal procedures were approved by the ethics committee of the Experimental Animal Center at our hospital, Ethical code: FUSCC‐IACUC‐S2022‐0358.

## CONSENT

Consent for publish has been obtained from the participant.

## Supporting information



Supporting Information

## Data Availability

All data generated and described in this article are available from the corresponding author on reasonable request.
