# Supplementary material for: PDPN+ cancer‐associated fibroblasts enhance gastric cancer angiogenesis via AKT/NF‐κB activation and the CCL2‐ACKR1 axis
Source: MedComm (2020). 2025 Jan 6;6(1):e70037. doi: 10.1002/mco2.70037 (PMC11702504; doi:10.1002/mco2.70037)
Supplement: Supplementary file 1 — Supporting Information [file MCO2-6-e70037-s001.docx]

Title page

Title: PDPN+ Cancer-Associated Fibroblasts Enhance Gastric Cancer Angiogenesis *via* AKT/NF-κB Activation and the CCL2-ACKR1 Axis

Authors: Zhenxiong Zhao^1,2#^, Hui Sun^2,3,4#^, Yingxue Liu^2,3,4#^, Yanqiu Zhang^2,5#^, Xin Wang^2,3,4^, Xu Wang^2,3,4^, Cong Tan^2,3,4^, Shujuan Ni^2,3,4^, Weiwei Weng^2,3,4^, Meng Zhang^2,3,4^, Lei Wang^2,3,4^, Dan Huang^2,3,4^, Wenchao Gu^6*^, Jinjia Chang^2,7*^, Weiqi Sheng^2,3,4*^, Mi-die Xu^2,3,4,##^

^1^ Department of Gastric Surgery, Fudan University Shanghai Cancer Center, Shanghai 200032, China.

^2^ Department of Oncology, Shanghai Medical College, Fudan University, Shanghai 200032, China.

^3^ Department of Pathology, Fudan University Shanghai Cancer Center, Shanghai 200032, China

^4^ Institute of Pathology, Fudan University, Shanghai 200032, China

^5^ Department of Endoscopy, Fudan University Shanghai Cancer Center, Shanghai, 200032, China

^6^ Department of Artificial Intelligence Medicine, Graduate School of Medicine, Chiba University, Chiba, Japan

^7^ Department of Medical Oncology, Fudan University Shanghai Cancer Center, Shanghai, 200032, China

**Running title:** Podoplanin-positive cancer associated fibroblasts in GC

^#^These authors contributed equally to this work.

^##^ Lead contact

^*^Corresponding Authors: Midie Xu (xumd27202003@sina.com); Weiqi Sheng (shengweiqi2006@163.com); Jinjia Chang (iamchangjinjia@163.com); and Wenchao Gu (sunferrero@gmail.com)

**
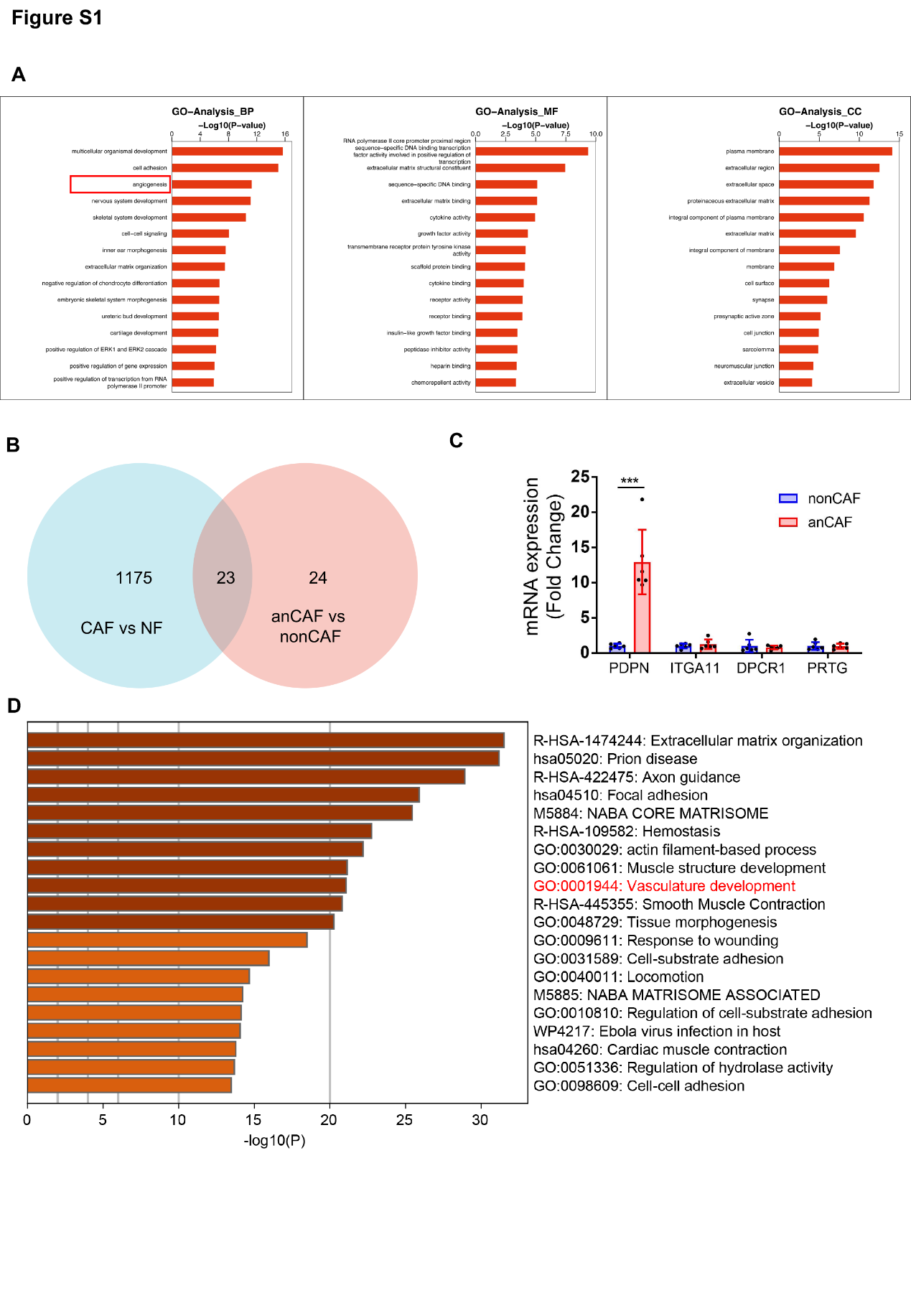
**

**Figure S1**

(**A**) Go Ontology analysis results showing differentially expressed genes between CAFs and NFs (n=12).

(**B**) Venn diagram illustrating the overlap of upregulated gene sets in Figure 1H and Figure 1I.

(**C**) qRT-PCR results showing the differential mRNA expression levels of the indicated cell-surface proteins in anCAFs and nonCAFs (n=6; ***p<0.001).

(**D**) Enrichment analysis revealed the biological process that highly expressed genes in PDPN+CCL2+CAFs were involved in.

**
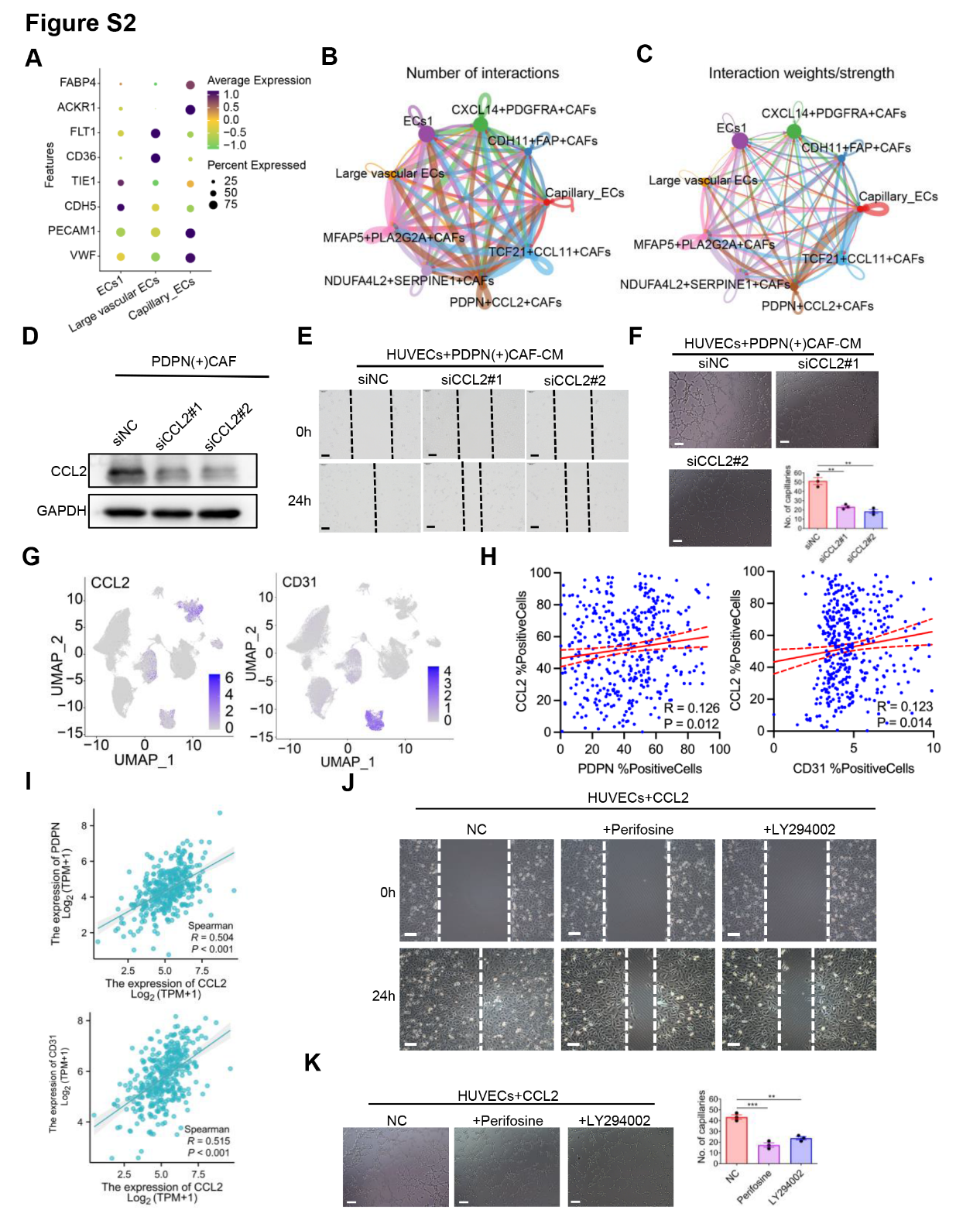
**

**Figure S2**

(**A**) Marker genes from each sub-cluster of endothelial cells are illustrated using a dot plot.

(**B-C**) Circle plots show the cellular interaction of CAFs and ECs subgroups in GC. CAFs and ECs were the core of the cellular interaction network (Edge width represents the numbers of interactions and node size represents the abundance of cell populations. The round loops along with cell type represent the interactions within the same cell type).

(**D**) Western blots results showing the expression level of CCL2 protein in indicated groups (n = 3).

(**E-F**) Wound healing assay (E) and tube formation assay (F) on HUVECs cultured in CM of PDPN(+)CAFs transfected with or without siRNA targeting CCL2. Scale bar = 100µm; **p<0.01.

**(G)** UMAP plots showing the expression of CCL2 and CD31 across the total cell population.

**(H)** Correlation analysis of CCL2 with PDPN and CD31 mRNA expression, as detected by IHC, in tumor tissue samples form 400 GC patients in the in-house dataset.

(**I)** Correlation analysis of CCL2 with PDPN and CD31 mRNA expression in tumor tissues of 407 GC patients in the TCGA dataset.

**(J-K)** Wound healing assay (J) and tube formation assay (K) on HUVECs treated with CCL2 with or without AKT inhibitors perifosine (10 µmol/L) and LY294002 (20 µmol/L). Scale bar = 100µm; **p < 0.01; ***p < 0.001.

**
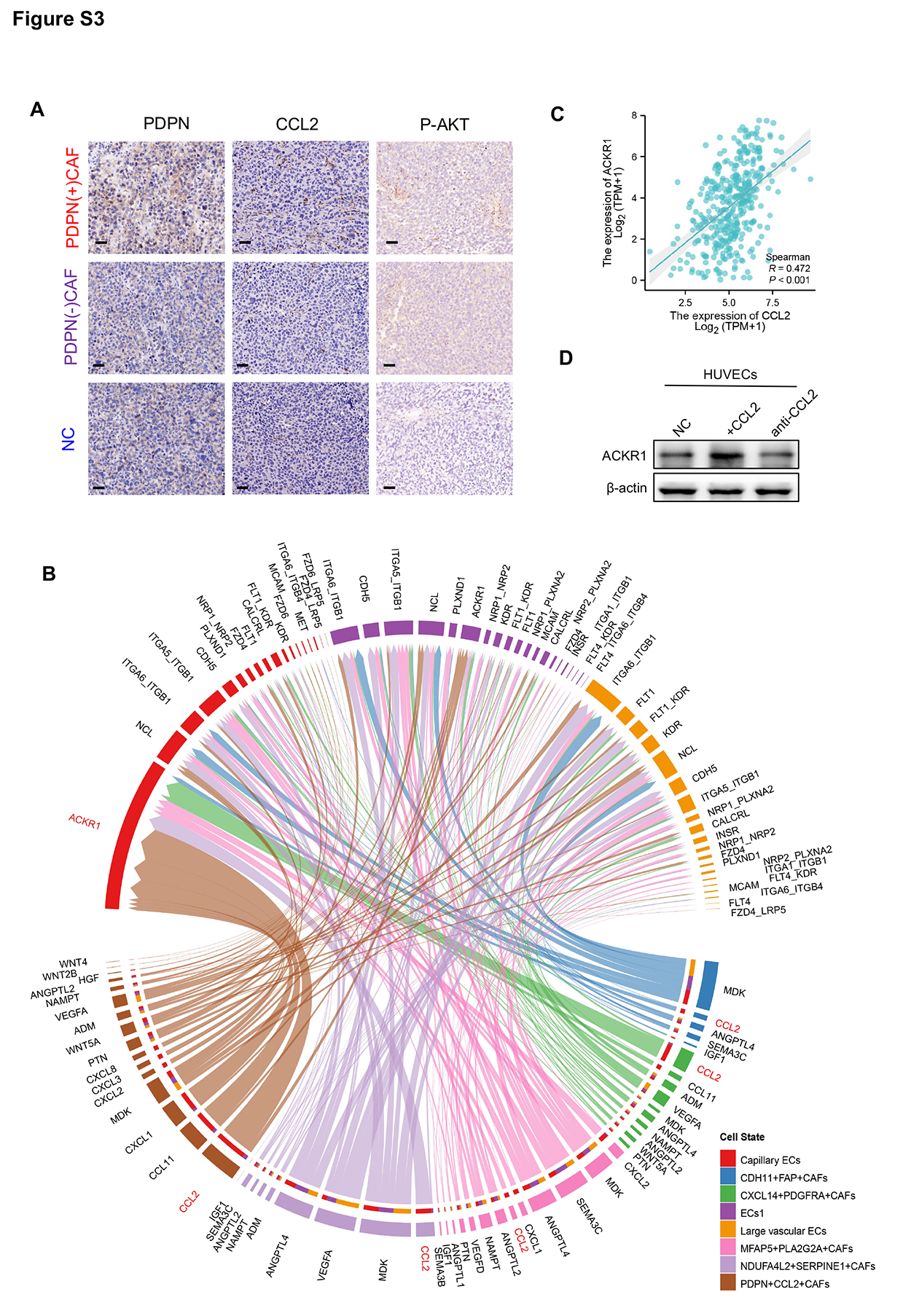
**

**Figure S3**

**(A)** Representative images showing IHC staining for PDPN, CCL2 and p-AKT in xenograft tumors harvested from the indicated groups. PDPN(+)CAF: MKN45 cells co-injected with PDPN(+)CAFs; PDPN(-)CAF: MKN45 cells co-injected with PDPN(-)CAFs; NC: MKN45 cells alone. Scale bar, 40 μm.

**(B)** Visualizing the ligand-receptor interactions between fibroblast and endothelial cell subsets using chord diagram, with CCL2 and ACKR1 specifically highlighted in red to underscore their significance within the network.

**(C)** Correlation of ACKR1 with CCL2 mRNA expression in tumor tissues of 407 GC patients in the TCGA dataset.

**(D)** Immunoblot showing ACKR1 expression in HUVECs treated with CCL2 or anti-CCL2.

**
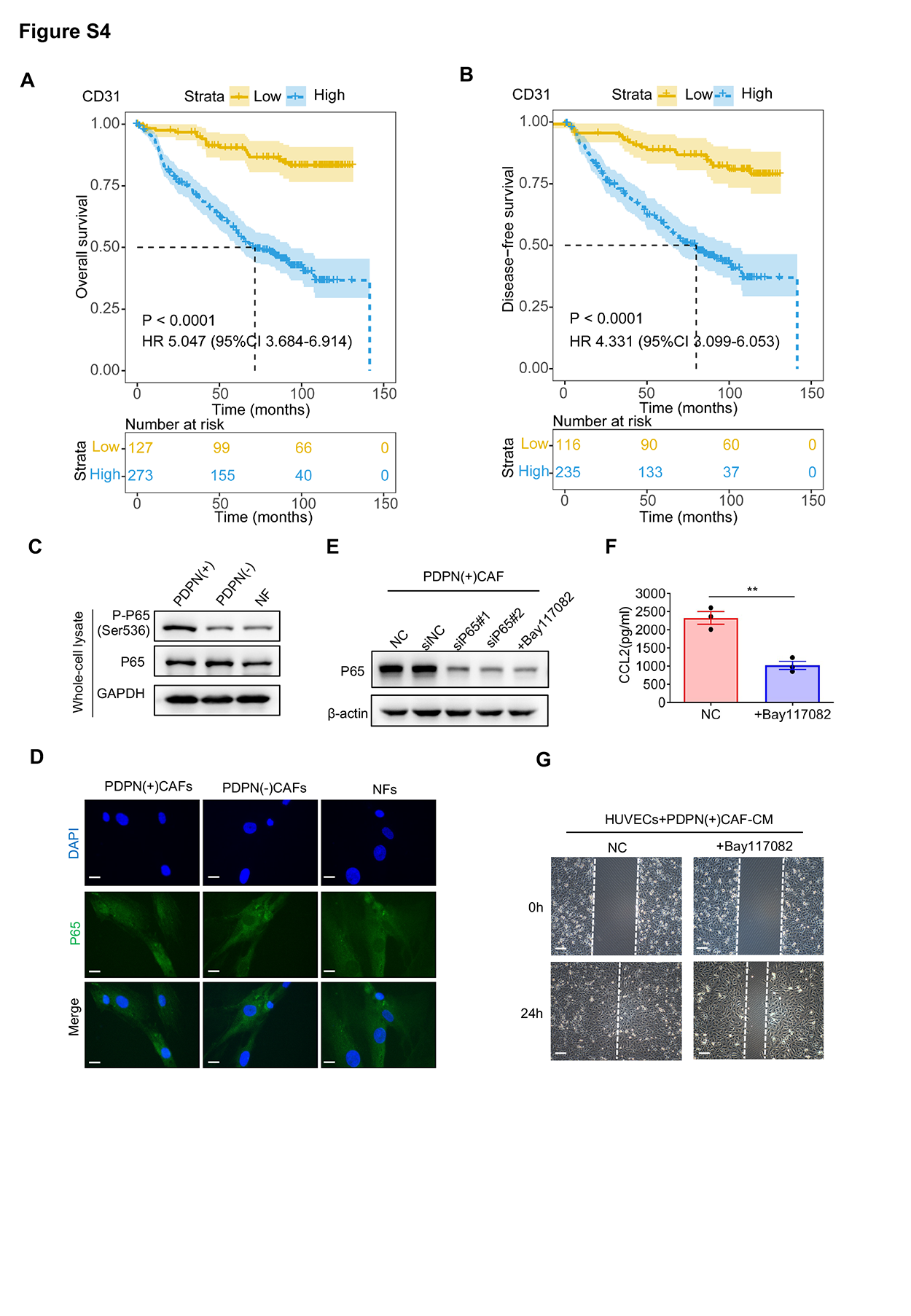
**

**Figure S4**

**(A-B)** Kaplan–Meier survival curves with log-rank analysis depicting overall survival (A) and disease-free survival (B) of GC patients based on the expression level of CD31 in cancer tissues in the in-house cohort.

**(C)** Immunoblot analysis of whole-cell p-P65 and P65 protein expression in PDPN(+)CAFs, PDPN(-)CAFs and NFs.

**(D)** Representative immunofluorescent staining of P65 in PDPN(+)CAFs, PDPN(-)CAFs and NFs (Scale bar = 25µm).

**(E)** Immunoblot analysis of P65 expression in PDPN(+)CAFs transfected with P65 siRNA, control siRNA or treated with NF-κB inhibitor Bay117082.

**(F)** ELISA results showing the amount of soluble CCL2 in CM from PDPN(+)CAFs treated with NF-κB inhibitor Bay117082 or DMSO (NC group) (**p < 0.01).

**(G)** Wound healing assay on HUVECs treated with CM from PDPN(+)CAFs treated with NF-κB inhibitor Bay117082 or DMSO (NC group) (Scale bar = 100µm; **p < 0.01).
